# Comparison of 5-Year Follow-up Outcomes Between Primary and Revision Roux-en-Y Gastric Bypasses After Open Vertical Banded Gastroplasty: an Inverse Propensity Score-Weighted Analysis

**DOI:** 10.1007/s11695-022-06189-4

**Published:** 2022-07-07

**Authors:** Mohamed Hany, Bart Torensma, Mohamed Ibrahim, Ahmed Zidan, Muhammad Gaballah, Ayman Farouk Mohammad Ahmed Aly, Ghada Ahmed Abu-Sheasha

**Affiliations:** 1 Department of Surgery, Medical Research Institute, Alexandria University, 165 Horreya Avenue, Hadara, 21561 Alexandria Egypt; 2 Bariatric Surgery at Madina Women’s Hospital (IFSO Center of Excellence), Alexandria, Egypt; 3 Leiden University Medical Center (LUMC), Leiden, The Netherlands; 4 Department of Bio-Medical Informatics and Medical Statistics, Medical Research Institute, Alexandria University, Alexandria, Egypt

**Keywords:** Roux-en-Y gastric bypass, Vertical banded gastroplasty, Bariatric surgery, Eating, Feeding behavior/physiology

## Abstract

**Introduction:**

Vertical banded gastroplasty (VBG) is associated with high weight regain; Roux-en-Y gastric bypass (RYGB) is used as a revision procedure in patients with VBG experiencing weight regain. This study compared the 5-year follow-up outcomes of primary (PRYGB) and revision RYGB after VBG (RRYGB).

**Methods:**

Patients who underwent PRYGB or RRYGB after VBG from 2008 to 2016 were enrolled. Data on weight regain, weight loss (WL), food tolerance (FT), early and late complications, and resolution or improvement in associated medical conditions were analyzed.

**Results:**

PRYGB and RRYGB groups had 558 and 156 patients, respectively, after exclusion of the lost to follow-up patients. PRYGB group showed significantly lower mean body mass index (over the entire follow-up period), early complications, reintervention rates for late complications, and overall reintervention rates than that of the RRYGB group. On the other hand, FT scores, odds of late complications, and improvements (in the fifth year) in associated medical conditions were comparable between the two groups.

**Conclusion:**

RRYGB in patients with VBG who regained weight showed comparable safety and resolution of associated diseases to that of PRYGB over the 5-year follow-up period. The WL in the RRYGB group was acceptable despite being less than that of the PRYGB group. FT was better after RRYGB than that of PRYGB in the first year; however, both were comparable at the fifth year follow-up. Patients with VBG undergoing RYGB should receive attentive treatment and evaluation of associated factors.

**Graphical abstract:**

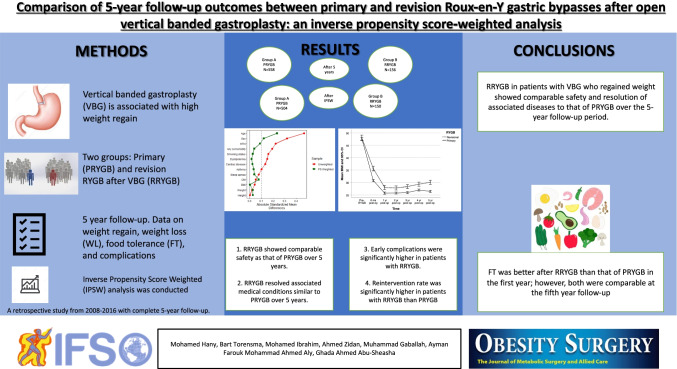

## Introduction

Vertical banded gastroplasty (VBG), after discovery in 1982, gained popularity and remained the preferred bariatric surgery gained popularity in the 1980s and the 1990s, and for a while, VBG was the preferred bariatric surgery in the USA [1, 2]; however, the method was associated with insufficient weight loss and weight regain, and are common after VBG, together with symptoms related to obstruction, such as gastroesophageal reflux disease and dysphagia [2, 3]. In most cases, weight regain post-VBG could be attributed to staple line disruption and patients consuming calorie-rich liquids and soft diets [2]. The Swedish Obese Subjects study reported a decline to 16% (from 25% after 2 years) in the total weight loss (%TWL) after 10-years post VBG [4], with a revision surgery rate of 28.3% after a mean follow-up period of 19 years [5]. Furthermore, revision surgery rates up to 50% have also been reported [3].

Roux-en-Y gastric bypass (RYGB), the bariatric surgery of recent times, is a commonly employed to revise failed bariatric procedures [6] because it can successfully manage the issues of weight regain and obstructive complications [2, 3]; however, revisions have been associated with lower outcomes in comparison to the primary procedures [7]. Revision surgery for failed VBG is still being performed, and more data about the outcomes of revision surgery will be of value for surgeons to take the best decisions*.* For instance, a previous systematic review has demonstrated that revision RYGB (RRYGB) shows lower outcomes when compared to the primary RYGB (PRYGB) [6]. Nevertheless, these systematic reviews showed mainly data from heterogenous studies in different settings. This study aimed to compare the 5 years follow-up outcomes between RRYGB (after VBG) and PRYGB performed in uniform settings.

## Methods

This retrospective study included patients who had PRYGB or RRYGB for failed VBG from 2008 to 2016 and rigorously completed the 5-year follow-up, at the Medical research institute, Alexandria University, and Madina women’s hospital IFSO-certified bariatric center.

The study was approved by the appropriate ethics committee and was performed in accordance with the ethical standards of the 1964 Declaration of Helsinki. All patients provided informed consent for the data being used for research publication.

### Study Endpoints

The primary endpoints were weight loss and the occurrence of early and late complications, while the secondary endpoints were the resolution of associated medical conditions and food tolerance (FT).

### Inclusion and Exclusion Criteria

Weight regain was defined as an increase in body weight reported by the patients after the previous VBG [8]. Those with VBG complications other than weight regain such as mesh erosion, mesh strictures, and abnormal laboratory nutritional markers, such as hypoalbuminemia, were excluded.

### Data Collection

Data on patients’ demographics, associated medical conditions at the time of operation, operative time, concomitant procedures (laparoscopic cholecystectomy, incisional hernias, and hiatal hernias), and hospital stay were obtained.

### Pre-operative Examinations for Revisions

Radiological (multi-detector computed tomography [MDCT] with virtual gastroscopy) and abdominal ultrasonography (U/S) examinations were performed. Findings of endoscopic examination performed before RRYGB were evaluated. FT was assessed using a one-page questionnaire (score between 1 and 27) [9].

### Pre-operative Examinations for Primary Cases

Routine laboratory tests, tests for macro-and micronutrients, findings of abdominal U/S examination, and echocardiography were assessed.

### Post-Operative Follow-ups After RYGB

All parameters were assessed included the early (within the first 30 days) and late (later than 30 days) complications, reoperations and readmissions, endoscopic findings, and resolution/improvement of associated medical conditions.

### Surgical Technique

The RRYGB and PRYGB group’s surgeries were performed by two independent surgeons (who operate on ~ 800 patients per year) as per the standard protocols and international guidelines (Appendix I).

### Post-operative Care

An oral liquid diet was started 12 h postoperatively. Enoxaparin was used for prophylaxis against thrombosis, starting 12 h preoperatively and continuing 24 h postoperatively for 21 days. Oral gastrografin series was routinely performed on day 1. All patients were prescribed multi-vitamin supplements, iron, and calcium citrates for life starting from week 3. Postoperative MDCT with oral and intravenous contrast was performed in patients with persistent abdominal pain, fever, tachycardia, obstructive symptoms, absolute constipation, and persistent vomiting.

### Statistical Methods

Continuous normally distributed and categorical variables were summarized using mean (M)/standard error (SE) and numbers (*n*)/percentages (%), respectively. To balance the baseline patient characteristics, propensity scores (PS)-based Inverse Propensity Score-Weighted (IPSW) analysis was conducted; the PS were first calculated using logistic regression, which included age, sex, initial height, weight, and BMI, presence of any associated medical conditions such as hypertension (HTN), diabetes mellitus (DM), obstructive sleep apnea, dyslipidemia, asthma, or cardiac disease, and smoking status. The IPSW was derived from the PS and used to weight the entire study sample.

The impact of IPSW analysis on the sample size was evaluated by calculating the effective sample size [10]. The standardized mean differences (*SMD*) of the baseline characteristics were calculated to ascertain the balance between the two cohorts. For correcting residual imbalances (*SMD* > 0.1), a double adjustment regression analysis, which included all the unbalanced covariates, was used. This approach was shown to be successful in removing the residual confounding bias [11]. Furthermore, we employed logistic, linear, and ordinal regressions to compare binary, continuous, ordinal outcomes, and results were expressed as odds ratio (OR), mean difference, and proportional OR with 95% confidence intervals (CI), respectively. Generalized Estimated Equations (GEE) were used to adjust for correlations among the observations of the same participant [12]. Furthermore, the Huber–White method [13] was used to estimate the SE. Improvement in associated medical conditions at five years postoperatively was summarized using partial credit scoring [14]. Scores of 10, five, and zero were provided when the associated medical condition resolved, improved, and unchanged, respectively, and the average score per group summarized the changes in the associated medical conditions. Statistical significance was set at *p* ≤ 0.05. Statistical analyses were performed using IBM SPSS Statistics and the cobalt R package [15].

## Results

A total of 691 and 196 patients underwent PRYGB and RRYGB procedures, respectively at 3 specialized bariatric centers. Furthermore, 558 (80.8%) and 156 (79.6%) patients in the PRYGB and RRYGB groups completed the 5-year follow-up, respectively, while the remaining were lost to follow-up with rates of 19.2% % in the PRYGB and 20.4% in the RRYGB.

### Baseline

**Fig. 1:**
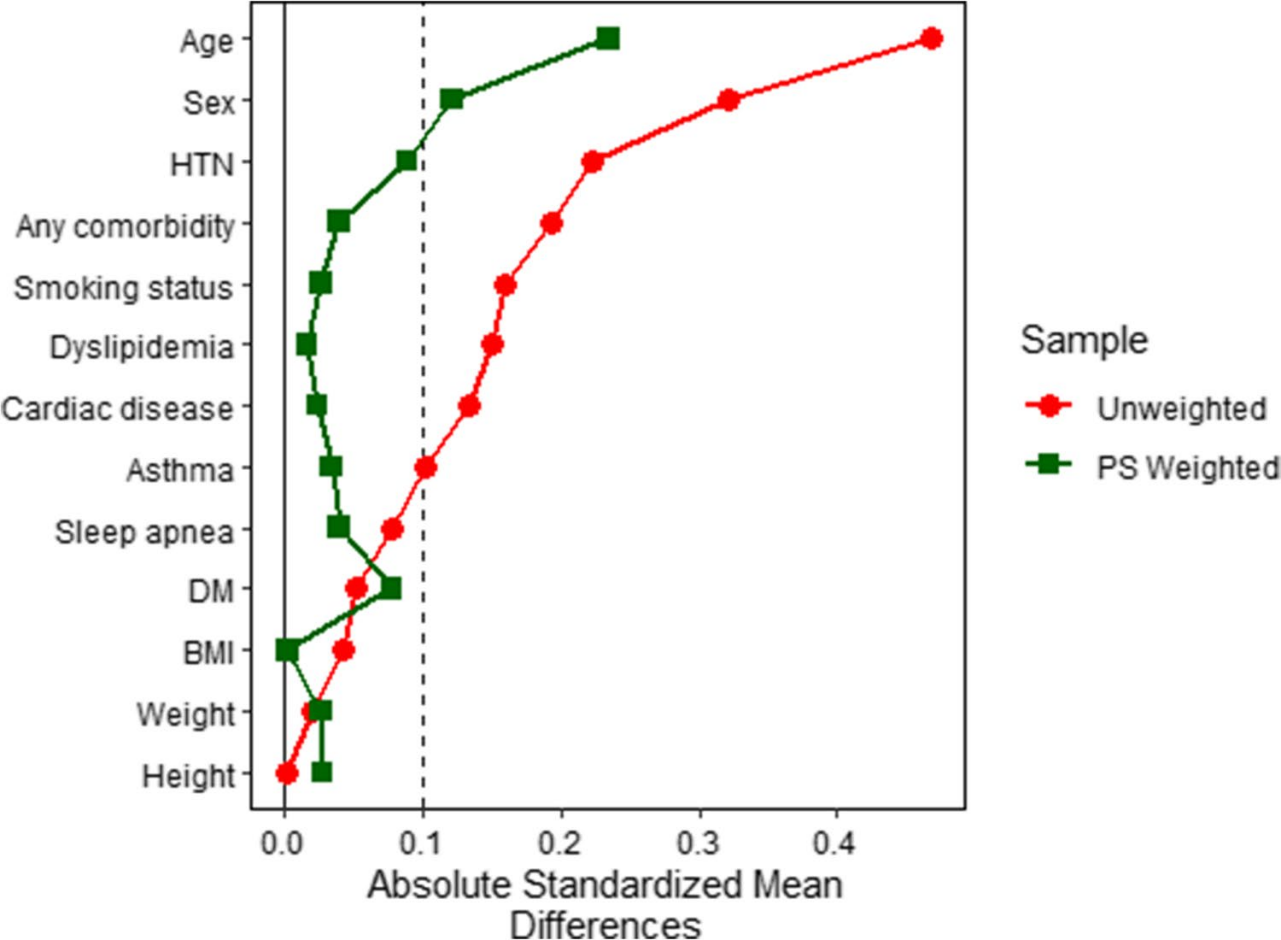
Absolute standardized mean differences in unweighted and weighted samples. A vertical line was superimposed denoting a standardized difference of 0.1, which is a threshold below which any imbalance is negligible

Before IPSW, the PRYGB patient group was significantly younger and had more patients with associated medical conditions (especially hypertension (HTN)) than that of the RRYGB group. No significant differences were observed in the distribution of sex, other associated medical conditions, or anthropometric measures between the two groups (Table 1). After IPSW, except age and sex, all other baseline covariates showed *SMD* < 0.1, improving balance, and without affecting the power of the study (effective sample size was 504 and 150 for the PRYGB and RRYGB groups, respectively (Fig. 1)). Before weighting, more than 0.01 *SMD* were observed in eight out of the 13 baseline covariates. With the IPSW, only age and sex had more-than-0.10 *SMD*. The balance was improved on almost all variables after adjustment, bringing all but two *SMD* < 0.1. The power of the study was also preserved as the “effective sample size” (*n* = 504 and *n* = 150 in the PRYGB and RRYGB) respectively) was like that of the original cohorts (Fig. 1).

**Table 1:** Comparison of baseline characteristics, associated medical conditions, and anthropometric measures between PRYGB and RRYGB before and after inverse propensity score weighting

	Unadjusted						Adjusted by IPSW					
	PRYGB		RRYGB		*P*	*aSMD*	PRYGB		RRYGB		*P*	*aSMD*
	n = 558		n = 156				n = 504		n = 150			
Age	38.4	(0.4)	42.9	(0.6)	(< .001)	0.539	39.4	(0.4)	41.7	(0.5)	(< .001)	0.270
Male	116	21%	42	27%	(.103)	0.362	133	26%	28	19%	(.271)	0.137
Associated medical conditions												
* Patients with one or more* *Associated medical condition*	356	64%	85	54%	(.034)	0.190	344	68%	95	63%	(.691)	0.039
* HTN*	168	30%	31	20%	(.012)	0.238	157	31%	48	32%	(.439)	0.096
* Dyslipidemia*	116	21%	42	27%	(.103)	0.144	122	24%	32	21%	(.864)	0.016
* Sleep apnea*	99	18%	23	15%	(.379)	0.081	95	19%	23	15%	(.703)	0.042
* DM*	93	17%	29	19%	(.573)	0.050	93	18%	21	14%	(.384)	0.076
* Asthma*	44	8%	8	5%	(.241)	0.112	41	8%	12	8%	(.779)	0.038
* Cardiac diseases*	16	3%	1	1%	(.107)	0.170	13	2%	4	3%	(.878)	0.030
Smoking status	30	5%	14	9%	(.099)	0.140	34	6%	8	5%	(.749)	0.024
Anthropometric measures												
* Height (cm)*	166.7	(0.4)	166.7	(0.5)	(.969)	0.003	166.7	(0.4)	166.4	(0.4)	(.659)	0.034
* Weight (kg)*	133.0	(1.1)	133.6	(1.8)	(.818)	0.022	133.1	(1.1)	132.4	(1.9)	(.749)	0.029
* BMI*	47.6	(0.3)	47.9	(0.6)	(.637)	0.043	47.7	(0.3)	47.7	(0.6)	(.977)	0.003

Categorical variables are expressed as counts (%) Continuous variables are expressed as means and (standard errors); *IPSW*, inverse propensity score weighting; *PRYGB*, primary Roux-en-Y gastric bypass; *RRYGB*, revisional Roux-en-Y gastric bypass; *aSMD*, absolute standardized mean difference; *HTN*, hypertension; *DM*, diabetes mellitus; *BMI*, body mass index

### Preoperative Findings in VBG Cases

Radiology identified complete staple line disruption, minor staple line disruption, and hiatal hernia in ten (6.4%), four (2.6%), and 12 (7.7%) patients, respectively, in RRYGB group U/S examinations indicated calculous cholecystitis in 15 (9.6%) and 64 (11.5%) patients of the RRYGB and PRYGB groups, respectively. Furthermore, in the RRYGB, pre-operative endoscopic examinations confirmed the complete staple line disruption in ten (6.4%), minor staple line disruption in four (2.56%), and grade “A” reflux esophagitis (Los Angeles classification) in seven patients (4.67%) [16].

### BMI Changes

**Fig. 2:**
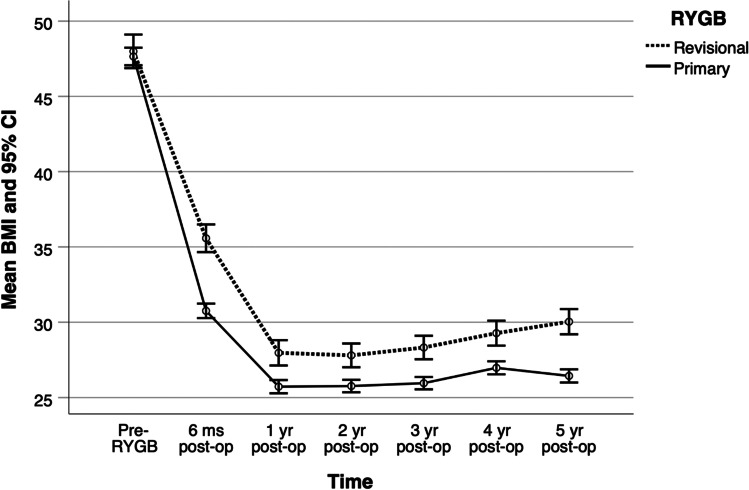
Changes in body mass index over the 5-year follow-up period

Patients of PRYGB group showed significantly lower mean BMI over the entire follow-up period than those of RRYGB group, particularly during the first postoperative 6 months (adjusted*MD*= 4.65; 95% CI 3.6 − 5.7;*p* ≤ 0.001). The rate of BMI decrease was higher in PRYGB (adjusted *MD*= 4.76; 95% CI 3.8 − 5.7) and RRYGB (adjusted*MD*= -2.8; 95% CI − 3.8 to − 1.8) groups during the first postoperative and following 6 months, respectively. Furthermore, in the next 3 years, BMI change was minimal in both the groups, while during the fifth year, a significant difference was noticed, with the PRYGB group showing a decrease of 0.5 BMI units, while the RRYGB group gaining 0.6 kg/m^2^(*p* ≤ 0.001) (Table 2 and Fig. 2).

**Table 2:** Comparisons of the BMI readings and change during the follow-up period between PRYGB and RRYGB

	PRYGB *n* = 504		RRYGB *n* = 150		Adjusted mean difference ${}^{a}$		*P*-value
	*M*	(SE)	*M*	(SE)	Est	(95% CI)	
BMI							
* Pre-operative*	47.7	(0.30)	47.7	(0.59)	− 0.02	(− 1.3, 1.3)	(.977)
* Post-operative*							
*1*^*st*^* 6 m*	30.5	(0.99)	35.2	(1.21)	4.65	(3.6, 5.7)	(< .001)
*2*^*nd*^* 6 m*	26.1	(1.13)	27.9	(1.30)	1.84	(1, 2.7)	(< .001)
*2*^*nd*^* year*	24.4	(1.05)	26.1	(1.16)	1.69	(0.9, 2.5)	(< .001)
*3*^*rd*^* year*	25.2	(1.16)	27.1	(1.29)	1.88	(1.1, 2.6)	(< .001)
*4*^*th*^* year*	26.5	(0.97)	28.2	(1.12)	1.70	(0.8, 2.6)	(< .001)
*5*^*th*^* year*	26.1	(0.94)	28.9	(1.11)	2.76	(1.8, 3.8)	(< .001)
Change in BMI							
*1*^*st*^* 6 m*	− 16.3	(1.55)	− 11.6	(1.69)	4.76	(3.8, 5.7)	(< .001)
*2*^*nd*^* 6 m*	− 4.4	(1.44)	− 7.2	(1.62)	− 2.81	(− 3.8, − 1.8)	(< .001)
*2*^*nd*^* year*	− 1.7	(1.29)	− 1.9	(1.37)	− 0.17	(− 0.8, 0.5)	(.605)
*3*^*rd*^* year*	0.8	(1.37)	1	(1.43)	0.19	(− 0.4, 0.8)	(.509)
*4*^*th*^* year*	1.3	(1.13)	1.2	(1.23)	− 0.18	(− 0.8, 0.4)	(.542)
*5*^*th*^* year*	− 0.5	(0.22)	0.6	(0.26)	1.07	(0.8, 1.4)	(< .001)

${}^{a}$ Adjusted for age and sex using multiple linear regression estimated using the generalized estimation equation and weighted by inverse propensity score weights where primary RYGB is the reference group; *PRYGB*, primary Roux-en-Y gastric bypass; *RRYGB*, revisional Roux-en-Y gastric bypass; *BMI*, body mass index; *M*, mean; *SE*, standard error; *Est.*, estimated value

### Food Tolerance

The mean FT score was higher in the RRYGB and PRYGB groups during the first and second postoperative years, respectively, and therefore, the FT scores were the whole period non-significant (*p* = 0.862) (Table 3).

**Table 3:** Comparisons of the FT score readings and changes during the follow-up period between PRYGB and RRYGB

	PRYGB *n* = 504		RRYGB *n* = 150		Adjusted mean difference ${}^{a}$		*P*-value
	M	(SE)	M	(SE)	Est	(95% CI)	
FT score							
Post-operative							
1^st^ year	21.4	(0.09)	22.9	(0.14)	1.47	(1.3, 1.6)	(< .001)
2^nd^ year	23.0	(0.14)	23.1	(0.19)	0.02	(− 0.2, 0.2)	(.862)
3^rd^ year	24.1	(0.14)	24.2	(0.19)	0.08	(− 0.1, 0.3)	(.412)
4^th^ year	24.1	(0.16)	24.2	(0.22)	0.04	(− 0.2, 0.3)	(.778)
5^th^ year	23.6	(0.14)	23.7	(0.20)	0.05	(− 0.2, 0.2)	(.653)
Change in the FT score							
2^nd^ year	1.6	(0.18)	0.1	(0.26)	− 1.45	(− 1.8, − 1.1)	(< .001)
3^rd^ year	1.1	(0.21)	1.1	(0.27)	0.02	(− 0.3, 0.3)	(.919)
4^th^ year	0.1	(0.19)	0	(0.24)	− 0.03	(− 0.3, 0.2)	(.822)
5^th^ year	− 0.5	(0.23)	− 0.5	(0.34)	0.03	(− 0.4, 0.5)	(.900)

${}^{{\varvec{a}}}$ Adjusted for age and sex using multiple linear regression estimated using generalized estimation equation and weighted by inverse propensity score weights, where primary RYGB is the reference group; *FT*, food tolerance test; *M*, mean; *SE*, standard error; *Est.*, estimated value; *PRYGB*, primary Roux-en-Y gastric bypass; *RRYGB*, revisional Roux-en-Y gastric bypass

### Operative Time, Length of Stay, and Complications

Operative time was significantly shorter (average 2.3 h) for the PRYGB group than that of the RRYGB group(*MD*= - 137.5; 95% CI: − 147.1 to − 127.8), while the length of hospital stay was equivalent between the two cohorts(*p* = 0.060). Although early complications were significantly lower in the PRYGB group (adjusted*OR*= 0.47; 95% CI 0.25 − 0.89), late complications were comparable between the two cohorts. Furthermore, leak occurred in one patient of each group(*p* = 0.33) (Table 4). Postoperative bleeding, requiring laparoscopic exploration in the first 2 days after surgery occurred in five and three patients with PRYGB (0.9%) and RRYGB (1.9%), respectively. Melena was encountered in nine (1.61%) and three (1.92%) patients with PRYGB and RRYGB, respectively. Marginal ulcers (MU), mainly characterized by melena and epigastric pain, were confirmed by endoscopy in 11 patients with PRYGB (1.97%) and 7 with RRYGB (4.49%)(*p* = 0.076). These 18 patients had risk factors such as smoking(*n* = 13), use of anti-inflammatory drugs(*n* = 4), or excessive alcohol consumption(*n* = 1), and were medically treated. Severe protein malnutrition occurred in one (0.18%) and four (2.56%)(*p* = 0.002) patients with PRYGB and RRYGB, respectively, and all required the laparoscopic reversal of the RYGB.

**Table 4:** Comparisons of operative time, hospital stay, and early and late complication between PRYGB and RRYGB

	PRYGB		RRYGB		Adjusted ${ES}^{a}$	*P*-value
	*n* = 504 *n*	%	*n* = 150 *n*	%		
Operative time (min)	42.6	(0.45)	180.1	(4.89)	***MD* = ** − 137.5 (− 147.1, − 127.8)	(< .001)
Hospital stay (day)	2.0	(0.01)	2.0	(0.01)	***MD* = *− ***0.03 (− 0.05, 0.001)	(.060)
Early complications	41	8.1%	22	14.7%	*OR* = 0.47 (0.25, 0.893)	(.021)
*UTI*	15	2.9%	6	4%		
*Pneumonia*	3	0.59%	3	2%		
*Wound infection*	2	0.39%	1	0.67%		
*Melena*	9	1.79%	3	2%		
*Bleeding from port site*	4	0.79%	1	0.67%		
*Bleeding from omentum*	1	0.19%	2	1.33%		
*MVO*	4	0.79%	3	2%		
*DVT*	1	0.19%	1	0.67%		
*Leakage*	1	0.19%	1	0.67%		
Late complications	61	12.1%	21	14%	***OR* = **0.77 (0.42, 1.403)	(.393)
*CCC*	43	8.5%	8	5.3%		
*Marginal ulcer*	11	2.18%	7	4.67%		
*Protein malnutrition*	1	0.19%	4	2.67%		
*Internal hernia*	3	0.59%	2	1.33%		
*Port site hernia*	4	0.79%	1	0.67%		

Categorical variables are expressed as counts (%). Continuous variables were expressed as means and (standard errors); ${ES}^{a}$, effect size adjusted for age and sex estimated using the generalized estimation equation (GEE) and weighted by inverse propensity score weights (IPSW) in multiple linear regression to quantify the mean difference (*MD*) and multiple logistic regression to estimate the odds ratio *(OR)*; *PRYGB*, primary Roux-en-Y gastric bypass; *RRYGB*, revisional Roux-en-Y gastric bypass; *UTI*, urinary tract infection; *MVO*, mesenteric vascular occlusion; *DVT*, deep vein thrombosis; *CCC*, chronic calculous cholecystitis

Internal hernias and port site hernias were observed in both the groups, and required laparoscopic repair. Re-intervention for late complications was required in eight and seven patients of the PRYGB and RRYGB groups, respectively; the rates being significantly higher in the RRYGB group (*p* = 0.019). Furthermore, the overall re-intervention rates were 2.3% and 6.4% in the PRYGB and RRYGB groups, respectively, and the RRYGB group had significantly higher rates for early and late complications (*p* = 0.012). Readmissions were recorded in 54 (9.68%) and 13 (8.3%) patients in the PRYGB and RRYGB groups, respectively.

### Associated Medical Conditions

Both cohorts demonstrated considerable improvements in diabetes mellites (DM) during the first and fifth years (*p* = 0.099). The odds of improvement of HTN were higher in the PRYGB (adjusted *OR* = 2.92; 95% CI 1.09 − 8.85) during the first year; however, both cohorts demonstrated a comparably high improvement in the fifth year. Dyslipidemia had considerable improvement in both cohorts during the first year. However, in the fifth year, the odds of improvement in the RRYGB cohort were significantly lower (*p* = 0.006). The resolution of associated medical conditions was defined according to international guidelines [17, 18] (Table 5).

**Table 5:** Comparison of the early and late impacts of PRYGB and RRYGB on main associated medical conditions

	*n* = *total*		No change		Improved		Resolved			${Score}^{a}$	*Adj. prop* ${OR}^{b}$ *(95% CI)*	*P*
			n	%	n	%		n	%			
Hypertension												
1^st^ year												
PRYGB	157	0	0%	21	13%	136		87%	9.33	2.92 (1.09, 7.85)		(.033)
RRYGB	48	0	0%	15	32%	33		68%	8.40			
5^th^ year												
PRYGB	157	0	0%	16	10%	141		90%	9.50	2.90		(.099)
RRYGB	48	4	8%	7	14%	37		76%	8.35	(0.82, 10.26)		
Dyslipidemia												
1st year												
PRYGB	122	0	0%	25	20%	97		80%	8.98	1.13 (0.46, 2.78)		(.795)
RRYGB	32	0	0%	7	22%	25		78%	8.96			
5th year												
PRYGB	122	0	0%	12	10%	110		90%	9.50	7.91 (1.83, 34.26)		(.006)
RRYGB	32	8	25%	4	13%	20		62%	6.88			
DM												
1st year												
PRYGB	93	0	0%	32	35%	61		65%	8.27	0.64 (0.23, 1.78)		(.392)
RRYGB	21	0	0%	5	24%	16		76%	8.82			
5^th^ year												
PRYGB	93	0	0%	19	20%	74		80%	8.99	1.68 (0.41, 6.88)		(.466)
RRYGB	21	3	12%	2	11%	16		77%	8.21			

${}^{{\varvec{a}}}$ Score of improvement estimated by partial credit score method; *Adj. prop. OR: proportional odds ratio a*djusted by age and sex using multiple ordinal regression estimated using generalized estimation equation and weighted by inverse propensity score weights, where the primary RYGB is the reference category; *DM*, diabetes mellitus; *PRYGB*, primary Roux-en-Y gastric bypass; *RRYGB*, revisional Roux-en-Y gastric bypass

## Discussion

### Outcomes of the Study

This study demonstrated that patients with PRYGB had significantly lower BMI than that of patients with RRYGB during the entire follow-up period; since regression adjustments were reported to remove residual confounding bias, this estimated difference in BMI was attributed only to surgical intervention [11]. Multiple large published series of laparoscopic RRYGB after failed VBG reported means of BMI from 28.6 to 28.8, at 9 years, and %TWL of 17.4% after a median of 74.3 months of follow-up, and %EWL ranging from 47 to 64.3% at 2 years after revision [19–23].

The results of this study were consistent with previous reports suggesting that RYGB induces up to 75% of EWL, with the best weight loss encountered at 18–24 months postoperatively. Minimal weight regain is observed in up to 50% of the patients after 2 years postoperatively [23]. Since distorted anatomy may interfere with certain steps, dissections, or neurohormonal changes, special attention to the weight changes in patients over time is necessary, especially after RRYGB. This study showed early higher mean FT scores after RRYGB in comparison to PRYGB, while at 5 years of follow-up, the FT scores were identical in both the groups. PRYGB was reported to have the worst early FT scores when compared to adjustable gastric banding and sleeve gastrectomy [24] and were mainly attributed to dumping and adaptation to the new surgery and the adaptation of the intestine to high-osmolarity food, with lower dumping symptoms [24, 25]. FT is an essential follow-up tool in training how to handle eating. Since VBG revision includes anatomical changes with loss of the pure restriction, it may lead to maladaptive eating and increased food passing and possible weight regain as a focus point.

PRYGB and RRYGB showed considerable improvement in the associated medical conditions throughout follow-up. Data from a meta-analysis showed no significant differences in DM and HTN resolution between PRYGB and RRYGB in 7 studies [6]. Reported resolution rates of associated medical problems in the large series of RRYGB after VBG are close to the rates of this study with reported DM resolution rates ranging from 57.1 to 75.7% and an improvement rate of 28.5%, with similar data for HTN and dyslipidemia [19, 22]. The main indications for readmission were vomiting and dehydration. This coincides with a previous report showing nausea/vomiting as a leading cause of readmission, followed by abdominal pain, and dehydration at rates of 12.95, 11.75, and 10.54%, respectively [26].

### Complications

Our data on complications were consistent with previous reports that showed higher complication rates in the RRYGB group than that in the PRYGB group [7]. Systematic reviews showed higher rates of complications (18.6% vs. 8.6%), leakages (4.3% vs. 1.39%), and mortality (0.6% vs. 0.2%) in patients with RRYGB vs. PRYGB [6], and RRYGB was also reported with a late complication rate of 11.8 − 14.2% [19, 20].

This study had one leak case in each group, with leak rates of 0.18% and 0.64% in the PRYGB and RRYGB respectively, comparable with the reported overall leak rate of 0.6% after RYGB [27]. One case in our study was successfully managed with percutaneous drainage alone, while the other case had Self Expandable Metallic Stent (SEMS). The timing of stenting is a factor influencing success; the earlier the intervention with stents, the higher the success rate [28–30]. Systematic reviews showed SEMS success rates of 73% and 76.1% for leaks after sleeve gastrectomy and RYGB, respectively [31].

In our cohorts, bleeding was the only indication for early reoperation, which was performed in 5 (0.9%) and 3 (1.9%) patients with PRYGB and RRYGB, respectively. Our findings are in agreement with other studies that reported bleeding as the leading cause of early surgical re-approach after bariatric surgery [32, 33].

Among late complications, MU was observed in both groups; MU is a common complication after RYGB, with an incidence of 1–16% [34]. Smoking, steroids, and NSAIDs are known risk factors for MU; however, MU can still occur without known risk factors and can be treated well [34, 35].

Severe protein malnutrition was diagnosed in one (0.18%) PRYGB, and four (2.56%) RRYGB patients, using the criteria of albumin levels < 2.5 g/dL from the literature [36]. The reported rates of protein malnutrition after RYGB ranged from 5 to 13% [36]. Laparoscopic reversal of the RYGB was done in all cases by dismantling the gastrojejunostomy and performing a side-to-side gastro-gastric anastomosis between the pouch and remnant stomach. According to widely reported prior knowledge, a length of at least 3 m of common channel should be checked before excising the alimentary limb [37].

Malnutrition and marginal ulcers are the most common causes of the reversal of RYGB [38]. Despite the low incidence in our study and the higher incidence described in the literature, good attention to this complication remains necessary with adequate therapy.

Internal hernias occurred in 3 (0.54%) and 2 (1.28%) patients with PRYGB and RRYGB, respectively, after closing the mesenteric defects. Furthermore, port-site hernias occurred in 4 (0.72%) and 1 (0.64%) patients with PRYGB and RRYGB, respectively, and all required surgical repair. Hernias are a common complication of RYGB with reported rates of 5.3% for internal hernias and 3.1% for trocar site hernias in large RRYGB for VBG [19, 21]. The incidence rate of internal hernias after PRYGB is reported to be up to 3.9% when not closing the mesenteric defects, and up to 1.3% when closing the defects, while some authors reported an incidence rate of 3.9% despite mesenteric defect closure with non-absorbable sutures [19, 39].

Although we detected significantly higher reintervention rates (6.4%) in the RRYGB group, even higher (7.1 to 12.4%) rates were reported previously [19–21, 39].

### Limitations

This study included a sufficient number of patients over a long period of time (5 years), however, had some limitations. First, we may have missed newer evidences due to retrospective nature of the study; therefore, a hidden bias and residual confounding may still be present even after using the propensity score and regression analysis to adjust for measured confounders, as unknown and known-but-not-measured variables were not considered. Second, data on exact weight loss and regain after primary VBG was not available, and hence, patients’ responses (positive or negative) to previous surgery were not clear. Third, laboratory nutritional assessment records were not available for all patients. Finally, another limitation was the use of the partial credit score [14]; this scoring obscures the details of changes in associated medical problems and assumes an equal interval between different changes in the same associated medical problems. Here, we did not use it to formally compare the improvement between the two cohorts as it was only used to summarize the change into an easily interpretable number.

## Conclusion

RRYGB is a demanding procedure that needs an experienced surgeon to achieve acceptable outcomes. RRYGB has lower but acceptable weight loss compared to PRYGB with comparable safety and resolution of associated medical diseases. FT was better in the RRYGB in the early follow-up, but later became comparable between RRYGB and PRYGB.

## Appendix 1: Surgical Techniques

### Revisional RYGB

The port sites are modified to avoid the expected areas of adhesions, pneumoperitoneum was created using visual entery trocar at the left mid-clavecular line about 10 cm below the costal margin, this port would be used as a right-hand working port. In case of adhesions with anterior abdominal wall hindering the insertion of the camera and left working ports, the assistant’s port in the left anterior clavicular line was first inserted to dissect these adhesions.

After creation of the pneumoperitoneum, adhesions between the stomach and liver were dissected using the energy device Enseal (Ethicon Endo-Surgery, Cincinnati, OH, USA) to identify the mesh to create the correct plane, then dissection continued up to the angle of His to identify the vertical staple line and to to expose the gastric serosa. The omentum was dissected off the greater omentum at the body and fundus, with division of the short gastric vessels using the energy device to free the whole fundus and body followed by dissection of the esophago-gastric junction with crural repair incase hiatal hernia was identified using 2/0 V-Loc non-absorbable sutures (Covidien, Mansfield, MA, USA). Intra-operative endoscopy was done at this step to identify position of the mesh and the vertical staple line.

Exploration of the whole small bowel was attempted before the creation of the gastric pouch to exclude the presence of adhesions or short mesentery to ensure tension-free gastrojejunostomy.

The creation of the gastric pouch of RYGB started by opening a window at the lesser curvature 1–2 cm above the mesh paying attention not to injure the blood supply, followed by stapling horizontally using Echelon Flex Endopath 60-mm linear stapler (Ethicon Endo-Surgery, Cincinnati, OH, USA) using black reloads, and then continued up to the angle of His using black and green reloads, over a 40 fr bougie and keeping the staple line medial to the previous vertical staple line inside the VBG pouch. Resection of the gastric fundus and part of the body including the mesh and the previous staple line was performed in all cases using the same stapler with green reloads fired transversely just below the mesh.

The lengths of the bilio-pancreatic and alimentary limbs were 60-100 cm, and 100 cm respectively. The gastro-jejunostomy and the jejuno-jejunostomy were performed with the same stapler using blue and white reloads respectively, with hand-sewn closure of the gastrostomy and enterostomies using 3/0 V-Loc 180 sutures (Covidien, Mansfield, MA, USA), white reloads were used for division of the jejunum.

All staple lines reinforced with seromuscular continuous sutures using 3/0 V-Loc 180 sutures (Covidien, Mansfield, MA, USA) in all cases. The mesenteric defects were routinely closed using 3/0 V-Loc non-absorbable sutures (Covidien, Mansfield, MA, USA). in all cases.

Endoscopic examination was done again to visualize the mucosa of the pouch and the gastro-jejunostomy to assess the vitality of the tissues, exclude intralumenal bleeding, and to make a leak test by air under water seal. blue dye leak test for the gastro-jejunostomy was also performed after endoscopy in all cases. A drain was inserted in the left sub-phrenic space at the end of the procedure.

### Primary RYGB

Pneumoperitoneum was created using visual entery trocars and 0o angled lens, standard 5 ports were used including three 12-mm ports (for the camera, right and left working ports) and two 5-mm ports (for liver retraction and for the assistant). The pouch was created with the same linear stapler using gold and blue reloads. The lengths of the bilio-pancreatic and alimentary limbs were 60–100 cm, and 100 cm respectively, the same as the revisional RYGB. The gastro-jejunostomy and the jejuno-jejunostomy were performed in the same way as revisional RYGB.

All staple lines were also reinforced with the same technique as the revisional RYGB. The mesenteric defects were also routinely closed. Intra-operative endoscopy was not routinely done for primary RYGB. Blue dye leak test for the gastro-jejunostomy was also routinely performed. A drain was also routinely inserted in the left sub-phrenic space.

Evolution of the Surgical Technique.

We had some changes in the instruments and materials used in the surgical procedure with no changes regarding the technique and the steps of the procedures:

- We used the Harmonic ACE (Ethicon Endo-Surgery, Cincinnati, OH, USA) for dissection and vessel sealing before 2012, and shifted to the Enseal (Ethicon Endo-Surgery, Cincinnati, OH, USA) later than 2012.
- We started using barbed sutures at 2013, before this time, we used the non-absorbable sutures Prolene (Ethicon, Somerville, New Jersey) for closure of the mesenteric defects and enforcement of the staple line, Vicryl (Ethicon, Somerville, New Jersey) for closure of the gastrostomies and enterostomies after stapling, and used Ethibond (Ethicon, Somerville, New Jersey) for hiatal repairs.

## References

[1] Mason EE (1982). Vertical banded gastroplasty for obesity. Arch Surg.

[2] Teixeira JA, Ranev D, Borao FJ, Binenbaum SJ, Matharoo GS (2020). Revision of vertical banded gastroplasty. Revisional foregut surgery.

[3] Sanchez H, Cabrera A, Cabrera K, Zerrweck C, Mosti M, Sierra M (2008). Laparoscopic Roux-en-Y gastric bypass as a revision procedure after restrictive bariatric surgery. Obes Surg.

[4] Sjöström L, Narbro K, Sjöström CD, Karason K, Larsson B, Wedel H (2007). Effects of bariatric surgery on mortality in Swedish obese subjects. N Engl J Med.

[5] Hjorth S, Näslund I, Andersson-Assarsson JC, Svensson P-A, Jacobson P, Peltonen M (2019). Reoperations after bariatric surgery in 26 years of follow-up of the Swedish Obese Subjects Study. JAMA Surg.

[6] Pędziwiatr M, Małczak P, Wierdak M, Rubinkiewicz M, Pisarska M, Major P (2018). Revisional gastric bypass is inferior to primary gastric bypass in terms of short-and long-term outcomes—systematic review and meta-analysis. Obes Surg.

[7] Qiu J, Lundberg PW, Javier Birriel T, Claros L, Stoltzfus J, El Chaar M (2018). Revisional bariatric surgery for weight regain and refractory complications in a single MBSAQIP accredited center: what are we dealing with?. Obes Surg.

[8] Jimenez A, Casamitjana R, Flores L, Viaplana J, Corcelles R, Lacy A (2012). Long-term effects of sleeve gastrectomy and Roux-en-Y gastric bypass surgery on type 2 diabetes mellitus in morbidly obese subjects. Ann Surg.

[9] Suter M, Calmes J-M, Paroz A, Giusti V (2007). A new questionnaire for quick assessment of food tolerance after bariatric surgery. Obes Surg.

[10] Ridgeway G, McCaffrey D, Morral A, Burgette L, Griffin BA (2017). Toolkit for Weighting and Analysis of Nonequivalent Groups: a tutorial for the twang package.

[11] Nguyen T-L, Collins GS, Spence J, Daurès J-P, Devereaux P, Landais P (2017). Double-adjustment in propensity score matching analysis: choosing a threshold for considering residual imbalance. BMC Med Res Methodol.

[12] Guo S, Fraser MW. Propensity score analysis: statistical methods and applications: SAGE publications 2014.

[13] Huber PJ, editor Under nonstandard conditions. Proceedings of the Fifth Berkeley Symposium on Mathematical Statistics and Probability: Weather Modification; University of California Press: Berkeley, CA, USA; 1967.

[14] Masters GN (1982). A Rasch model for partial credit scoring. Psychometrika.

[15] Greifer N. cobalt: Covariate Balance Tables and Plots. R package, version 4.3.2. [2022] - [cited 2022 Feb 4]. Available from: https://CRAN.R-project.org/package=cobalt.

[16] Sami S, Ragunath K (2013). The Los Angeles classification of gastroesophageal reflux disease. Video J Encycl GI Endosc.

[17] Brethauer SA, Kim J, El Chaar M, Papasavas P, Eisenberg D, Rogers A (2015). Standardized outcomes reporting in metabolic and bariatric surgery. Obes Surg.

[18] Benaiges D, Sagué M, Flores-Le Roux JA, Pedro-Botet J, Ramón JM, Villatoro M (2016). Predictors of hypertension remission and recurrence after bariatric surgery. Am J Hypertens.

[19] Khewater T, Yercovich N, Grymonprez E, Horevoets J, Mulier JP, Dillemans B (2019). Twelve-year experience with Roux-en-Y gastric bypass as a conversional procedure for vertical banded gastroplasty: are we on the right track?. Obes Surg.

[20] Suter M, Ralea S, Millo P, Allé J-L (2012). Laparoscopic Roux-en-Y gastric bypass after failed vertical banded gastroplasty: a multicenter experience with 203 patients. Obes Surg.

[21] Gagné DJ, Dovec E, Urbandt JE (2011). Laparoscopic revision of vertical banded gastroplasty to Roux-en-Y gastric bypass: outcomes of 105 patients. Surg Obes Relat Dis.

[22] Sarhan MD, AbdelSalam NM, Mostafa MS, Yehia A, Anwar I, Fathy E (2021). Laparoscopic Roux-en-Y gastric bypass after failed vertical banded gastroplasty: 2-year follow-up of 102 patients. Obes Surg.

[23] Magro DO, Geloneze B, Delfini R, Pareja BC, Callejas F, Pareja JC (2008). Long-term weight regain after gastric bypass: a 5-year prospective study. Obes Surg.

[24] Overs SE, Freeman RA, Zarshenas N, Walton KL, Jorgensen JO (2012). Food tolerance and gastrointestinal quality of life following three bariatric procedures: adjustable gastric banding, Roux-en-Y gastric bypass, and sleeve gastrectomy. Obes Surg.

[25] Parkes E (2006). Nutritional management of patients after bariatric surgery. Am J Med Sci.

[26] Aman MW, Stem M, Schweitzer MA, Magnuson TH, Lidor AO (2016). Early hospital readmission after bariatric surgery. Surg Endosc.

[27] Mocanu V, Dang J, Ladak F, Switzer N, Birch DW, Karmali S (2019). Predictors and outcomes of leak after Roux-en-Y gastric bypass: an analysis of the MBSAQIP data registry. Surg Obes Relat Dis.

[28] Hany M, Ibrahim M, Zidan A, Samir M, Elsherif A, Selema M (2021). Role of primary use of mega stents alone and combined with other endoscopic procedures for early leak and stenosis after bariatric surgery, single-institution experience. Obes Surg.

[29] Murino A, Arvanitakis M, Le Moine O, Blero D, Devière J, Eisendrath P (2015). Effectiveness of endoscopic management using self-expandable metal stents in a large cohort of patients with post-bariatric leaks. Obes Surg.

[30] Quezada N, Maiz C, Daroch D, Funke R, Sharp A, Boza C (2015). Effect of early use of covered self-expandable endoscopic stent on the treatment of postoperative stapler line leaks. Obes Surg.

[31] Okazaki O, Bernardo WM, Brunaldi VO, Junior CC, Minata MK, de Moura DT (2018). Efficacy and safety of stents in the treatment of fistula after bariatric surgery: a systematic review and meta-analysis. Obes Surg.

[32] Augustin T, Aminian A, Romero-Talamás H, Rogula T, Schauer PR, Brethauer SA (2016). Reoperative surgery for management of early complications after gastric bypass. Obes Surg.

[33] Ladak F, Dang JT, Switzer NJ, Mocanu V, Birch DW, Karmali S (2019). Rates of reoperation and nonoperative intervention within 30 days of bariatric surgery. Surg Obes Relat Dis.

[34] Moon RC, Teixeira AF, Goldbach M, Jawad MA (2014). Management and treatment outcomes of marginal ulcers after Roux-en-Y gastric bypass at a single high volume bariatric center. Surg Obes Relat Dis.

[35] Kalaiselvan R, Exarchos G, Hamza N, Ammori BJ (2012). Incidence of perforated gastrojejunal anastomotic ulcers after laparoscopic gastric bypass for morbid obesity and role of laparoscopy in their management. Surg Obes Relat Dis.

[36] Kuin C, den Ouden F, Brandts H, Deden L, Hazebroek E, van Borren M (2019). Treatment of severe protein malnutrition after bariatric surgery. Obes Surg.

[37] Campos GM, Ziemelis M, Paparodis R, Ahmed M, Davis DB (2014). Laparoscopic reversal of Roux-en-Y gastric bypass: technique and utility for treatment of endocrine complications. Surg Obes Relat Dis.

[38] Ma P, Ghiassi S, Lloyd A, Haddad A, Boone K, DeMaria E (2019). Reversal of Roux en Y gastric bypass: largest single institution experience. Surg Obes Relat Dis.

[39] Torensma B, Kooiman L, Liem R, Monpellier VM, Swank DJ, Tseng L (2021). Internal herniation incidence after RYGB and the predictive ability of a CT scan as a diagnostic tool. Obes Surg.

